# Dietary diversity and associated factors among pregnant women attending antenatal care at public hospitals in North Shewa, Oromia, Ethiopia, 2023

**DOI:** 10.3389/fnut.2024.1400813

**Published:** 2024-09-25

**Authors:** Fikadu Tolesa Alemu, Adugna Alemu Desta, Asfaw Getaye Tola

**Affiliations:** ^1^Department of Midwifery, College of Health Sciences, Salale University, Fiche, Ethiopia; ^2^Department of Nursing, College of Health Sciences, Salale University, Fiche, Ethiopia

**Keywords:** pregnant women, food groups, dietary diversity, socioeconomic and sociodemographic factors, Ethiopia

## Abstract

**Background:**

Adequate consumption of diversified food during pregnancy enables adequate intake of 11 important micronutrients. Pregnant women who consume an inadequate amount of diversified food during pregnancy are at a higher risk of delivering preterm babies, stillbirth, low birth weight, and small for gestational age newborns.

**Objective:**

This study aimed to assess dietary diversity (DD) and associated factors among pregnant women attending antenatal care (ANC) at public hospitals in North Shewa, Oromia, Ethiopia, 2023.

**Methods and materials:**

An institution-based cross-sectional study was conducted on 400 pregnant women attending antenatal care. The study participants were selected using a systematic random sampling method. A pre-tested, structured, and interviewer-administered questionnaire was used to collect information about dietary diversity. The questionnaire was adapted from a validated and modified individual dietary diversity guideline, as recommended by the Food and Agriculture Organization (FAO) of the United Nations. The household food security level was determined using a standard set of questions derived from the Household Food Insecurity Access Scale (HFIAS) measurement guide. The data were entered into EpiData version 4.6 software and exported to Statistical Package for the Social Sciences (SPSS) version 24 for analysis. The results from the bivariate analysis of *p* < 0.25 were moved to the multivariable logistics regression analysis model. Any statistical test with a *p* < 0.05 at a 95% confidence interval (CI) was considered statistically significant.

**Results:**

The study revealed that 55.4% of the pregnant women had adequate dietary diversity. Being an urban dweller [adjusted odds ratios (AOR = 2.4. 95% CI: 1.1, 5.18)], being a housewife (AOR = 3.44, 95% CI: 1.37, 8.68), being employed (AOR = 3.69, 95% CI: 1.12, 12.16), being a merchant (AOR = 3.43, 95% CI: 1.15, 10.24), being a daily laborer (AOR = 3.66, 95% CI: 1.0, 13.45), having a low average monthly household income of <500 Ethiopian birr (ETB) (AOR = 0.25, 95% CI: 0.07, 0.83), practicing home gardening (AOR = 2.5, 95% CI: 1.39, 4.5), meal frequency being three or more times per day (AOR = 2.9, 95% CI: 1.64, 5.09), and receiving dietary counseling during antenatal care (AOR = 3.56, 95% CI: 2.0, 6.35) were factors associated with the dietary diversity of the pregnant women.

**Conclusion and recommendations:**

This study found that 55.4% of the pregnant women had adequate dietary diversity. Place of residence, women’s occupation, average monthly household income, practicing home gardening, meal frequency per day, and receiving dietary counseling during antenatal care were strong predictors of adequate dietary diversity among pregnant women. Therefore, attention should be paid to pregnant women’s diet by healthcare providers during antenatal care.

## Introduction

Dietary diversity (DD) is the consumption of a variety of foods across a number of different food groups over a given reference period, which enables the safeguarding of adequate intake of micronutrients (1). Dietary diversity (DD) quantifies the number of foods or food groups in a diet, which is a valid proxy indicator of the availability of dietary energy at the household level, the adequacy of micronutrients in the diets of young children and women of reproductive age, and is a predictor of nutritional status (1, 2). The foods or food groups that are included in the minimum dietary diversity of women (MDD-W) mostly reflect the diet quality, with the probability of the minimum micronutrient adequacy of women’s diets summarized across 11 important micronutrients, which are vitamin A, vitamin B1 (thiamine), vitamin B2 (riboflavin), vitamin B3 (niacin), vitamin B6, vitamin B12, vitamin C, folate, calcium, iron, and zinc (2).

A healthy diet during pregnancy should include adequate energy, protein, vitamins, and minerals, obtained through the consumption of a diversified diet, which includes green and orange vegetables, meat, fish, beans, nuts, whole grains, and fruits (3). A woman who has been well nourished before conception begins her pregnancy with reserves of several nutrients, which can help meet the recurrent needs of the growing fetus without adversely affecting her health. Infants who receive proper nourishment in the womb have an improved chance of entering life in very good health (4). There is a growing recognition of the significance of maternal nutrition interventions as part of antenatal care (ANC) (5), with high priority given to nutrition through dietary interventions and micronutrient supplementation. This coupled with health system interventions aimed at improving the use and quality of ANC (6).

Malnutrition is universally recognized as the most important risk factor for morbidity and mortality of hundreds of millions of pregnant women. Globally, in 2018, 9.7% (153.8 million) of women (aged 20–49) and 5.7% (16.2 million) of adolescent girls (aged 15–19) were estimated to be underweight and 32.8% (613.2 million) women of reproductive age and 35.3 million pregnant women were estimated to be anemic (7). In developing countries, dietary diversity is given greater importance, especially in addressing nutritional deficiencies; there is a widespread recognition that low dietary diversity is associated with chronic nutritional deficiencies (8). Ethiopia is no exception in this regard, as roughly one out of every four women aged 15–49 years is undernourished [body mass index (BMI) <18.5 kg/m^2^] (9). A lack of dietary diversity and micronutrient-dense food consumption is a particularly severe problem among Ethiopian women as cereals or grains contribute to the highest proportion of women’s diet, which are low in quality, diversity, and micronutrient content (10). In 2015, the adequate dietary diversity intake of adult women was found, in general, to be extremely low, as only 20.3% of the adult women consumed 5 or more food groups out of 10 food groups (11). Ethiopia scored the lowest among 187 countries for fruit consumption among women and second lowest (above Vanuatu) for vegetable consumption (12). In addition, compared to the WHO recommendations regarding vegetables and fruits consumption, the average Ethiopian vegetables and fruits consumption meets only 36.4% of the recommendation (13).

A diet dominated by staple foods with little diversity can contribute to the burden of micronutrient deficiencies and malnutrition, which is already high in Ethiopia (10). In a study, it was found that pregnant women who consumed an adequate amount of diversified foods during pregnancy had a low risk of maternal anemia, preterm delivery, and low birth weight (14). However, compared to adequate consumption, poor or inadequate consumption of dark green leafy vegetables, dairy products, and fruits and vegetables during pregnancy were associated was a higher risk of preterm delivery, stillbirth, and low birth weight (15). Pregnant women who consumed a diet with a low amount of grains, meat, dairy, fruits, and vegetables and had inadequate total diversity intake during the last trimester of pregnancy had a higher percentage of delivering a small for gestational age and low birth weight newborn (16).

Some studies have explored the factors influencing maternal dietary diversity adequacy in different parts of the country. For instance, sociodemographic and socioeconomic factors (17, 18), household food security status (19, 20), illness during pregnancy and dietary intake counseling during antenatal care (21), meal frequency per day (22), and various forms of taboos, misconceptions, and cultural beliefs toward certain foods during pregnancy (23, 24) were explored as predictors of maternal dietary diversity during pregnancy. Although the government of Ethiopia endorsed various intervention programs to address nutrition-related health problems, inadequate dietary diversity and nutritional status of pregnant women remain a prominent problem (25). Therefore, this study aimed to assess dietary diversity adequacy and the associated factors among pregnant women attending antenatal care at public hospitals in North Shewa, Oromia, Ethiopia, 2023.

### Study design and setting

An institutional-based cross-sectional study was conducted among pregnant women attending antenatal care at public hospitals in North Shewa, Oromia, Ethiopia, 2023. The North Shewa zone is located in the Oromia region, Ethiopia, 112 km northwest of the capital city, Addis Ababa. The zone has a total population of approximately 1,639,586, of which 717,552 and 922,034 are men and women, respectively, with a majority (89.75%) of the population residing in rural areas. North Shewa zone has four public hospitals, namely Salale University Comprehensive Specialized Hospital, Kuyu Hospital, Dera Hospital, and Muka Turi Hospital.

### Sample size and sampling procedure

The sample size was determined using a single proportion population formula, with the assumption that 38.5% of pregnant women consumed adequate dietary diversity, which was drawn from a previous study conducted in Alamata General Hospital, Ethiopia (26), along with a 95% confidence interval (CI), a 5% margin of error, and a 10% non-response rate. The final estimated sample size was 400. The study was conducted in four public hospitals, and the participants from each hospital were allocated proportionally after identifying the 3 months (September, October, and November 2022) of antenatal care follow-up of the pregnant women at each hospital. The study participants were selected using a systematic random sampling method. The sampling interval was calculated by dividing the monthly attendance for the antenatal care follow-up at each health facility by the calculated sample size. The calculated sample interval value for each hospital was two. The first study participant was selected using a lottery method, and it was continued at every two intervals at each hospital in a similar pattern until the required numbers of samples were met.

### Inclusion and exclusion criteria

Pregnant women attending ANC services who provided informed consent during the data collection period were included in the study. However, those involved in intervention programs (such as supplementary feeding or general food distribution), those unable to speak or hear, and those who were seriously ill during the data collection period were excluded from the study.

### Data collection tool

A pre-tested structured interviewer-administered questionnaire was used. A questionnaire for collecting information about the socio-demographic, socio-economic, pregnancy, and feeding pattern-related status was adapted and reviewed from a literature review (26–28). In addition, a questionnaire for collecting information regarding the dietary diversity of the pregnant women was adapted from a validated and modified individual dietary diversity guideline, as recommended by the Food and Agriculture Organization (FAO). The approach to collect data on dietary diversity was a qualitative 24-h recall of all the foods and drinks consumed by the respondents.

A minimum serving size for each food group was defined based on standard measurement units, such as units, cups (ounces), and a tablespoon (≥15 g), which were (approximated by women) assigned to a defined food group (2). The household food security level of the pregnant women was determined using a standard set of questions derived from the Household Food Insecurity Access Scale (HFIAS) measurement guide, which was developed by the Food and Nutrition Technical Assistance (FANTA)/United States Agency for International Development (USAID) (29). The questionnaire was carefully translated first into the local language, Afaan Oromo and Amharic, and then translated back to English for data analysis by a language expert. In addition, a human nutrition expert (holder of a master’s degree in human nutrition and working at Salale University, College of Health Sciences, Abebech Gobena Campus, as academic staff) was consulted to improve the local validation of the tool.

### Data quality control

The pre-test was conducted outside of the study area on 21 pregnant women attending ANC, and the questionnaire was assessed for its content, length, and word selection. This was helpful in modifying the questionaries by involving missed foods or by avoiding inapplicable foods in the study area. Training was given to the data collectors and the supervisor, who were trained for 2 days before the data collection on the whole data collection procedure. The procedure manual for the data collection was prepared and distributed to the data collectors and the supervisor. During the data collection, the principal investigator and the supervisor reviewed the filled questionnaires on a daily basis. Before the data entry, each questionnaire was given a unique code by the principal investigator.

### Data processing and analysis

The completed questionnaires were checked for completeness and were coded; the data were entered into EpiData 4.6 and exported to Statistical Package for the Social Sciences (SPSS) version 24 for analysis. The data were cleaned using frequencies and cross-tabulations. The description of the means, frequencies, and proportions of the given data for each variable was calculated. The variables with a *p* < 0.25 in the bivariate analysis were entered into the multivariable logistic regression analysis model to control for the effects of the confounders. A variable with a *p* < 0.05 at a 95% confidence interval was considered statistically significant. Crude and adjusted odds ratios (AOR) with their 95% confidence intervals were calculated. Finally, the results of the study were presented using tables, figures, and text based on the obtained data.

### Ethical considerations

Ethical clearance to conduct the study was obtained from Salale University, College of Health Sciences, Ethical Review Committee (Ref. number HSC/567/2023). An additional permission letter was granted from the North Shewa zone health bureau. Finally, after explaining the purpose and process of the research to the study participants, informed consent was obtained from each participant. To maintain the privacy of the respondents, their names and identities were not included in the data collection questionnaire.

## Results

In this study, a total of 395 pregnant women were involved, with a response rate of 98.75%.

### Socio-demographic characteristics

The mean ± standard deviation (SD) of the respondents’ age was 26.88 ± 4.94 years, and the majority, 149 (37.7%), of them were between 23 and 28 years of age. Most of the respondents, 264 (66.8%), were Orthodox Tewaheido Christian religion followers, and 343 participants (86.8%) were married. A total 346 (87.6%) of the pregnant women reported that they live in urban areas.

Around one-third of the participants, 97 (24.6%), reported that they could read and write. More than three-fourths, 297 (75.2%), of the households were headed by the husband and had no pregnant women household heads, and 112 (28.4%) heads of the households had elementary-level formal education. The majority of the study participants, 275 (69.6%), reported having <5 family members. More than half of the pregnant women were housewives, 224 (56.7%) (Table 1).

**Table 1:** Sociodemographic characteristics of the pregnant women attending antenatal care at public hospitals in North Shewa, Oromia, Ethiopia, 2023 (*n* = 395).

Variables	Frequency	%
Age		
17–22	95	24.1
23–28	149	37.7
29–34	125	31.6
35–40	26	6.6
Religion		
Orthodox	264	66.8
Protestant	64	16.2
Muslim	39	9.9
Others*	28	7.1
Marital status		
Single	14	3.5
Married	343	86.8
Widowed	22	5.6
Divorced	16	4.1
Residence		
Urban	346	87.6
Rural	49	12.4
Maternal educational status		
Unable to read and write	66	16.7
Able to read and write	97	24.6
Elementary school	81	20.5
Secondary and preparatory school	75	19
College and above	76	19.2
Head of the household		
Husband	297	75.2
Wife	74	18.7
Both	24	6.1
Head of the household educational status		
Unable to read and write	40	10.1
Able to read and write	51	12.9
Elementary school	112	28.4
Secondary and preparatory school	101	25.6
College and above	67	17
Number of family members		
Below five	275	69.6
Five and above	120	30.4
Mother’s occupation		
Housewife	224	56.7
Employed	60	15.2
Merchant	53	13.4
Daily laborer	27	6.8
Others**	31	7.8

*Catholics, traditional believers. **Students, unemployed, etc.

### Socio-economic characteristics

More than one-third, 142 (35.9%), of the study participants had trade and private enterprises as the main source of household income. Furthermore, slightly more than half of the participants, 266 (67.3%), had an average monthly income between 501 and 1,500 Ethiopian birr (ETB). Regarding the source of drinking water for the members of the pregnant women’s households, 362 (91.6%) of the mothers reported tap water as the main source of their drinking water. With regard to the presence of a latrine in their home, 354 (89.6%) of the pregnant women reported that they had a toilet in their home. Slightly more than one-third of the pregnant women, 128 (32.4%), reported that they practice home gardening (Table 2).

**Table 2:** Socio-economic characteristics of the pregnant women attending antenatal care at public hospitals in North Shewa, Oromia, Ethiopia, 2023 (*n* = 395).

Variables	Frequency	%
Main source of household income		
Farming and livestock	102	25.8
Trade and private enterprise	142	35.9
Employed	78	19.7
Daily laborer	48	12.2
Others	25	6.3
Average monthly income		
Below 500 ETB	24	6.1
501–1,500 ETB	266	67.3
Above 1,501 ETB	105	26.6
Source of drinking water		
Tap water	362	91.6
Pumping water	16	4.1
Protected well	17	4.3
Latrine present		
Yes	354	89.6
No	41	10.4
Home gardening practice		
Yes	128	32.4
No	267	67.6

### Pregnant women’s household food security status

**Figure 1:**
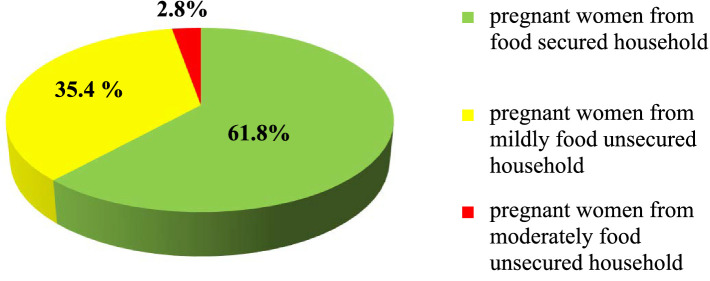
Household food security status of the pregnant women attending antenatal care at public hospitals in North Shewa, Oromia, Ethiopia, 2023.

More than half of the pregnant women, 244 (61.8%), were from food secured households, and 140 (35.5%) of the pregnant women were from mildly food unsecured households, where the members worried about not having enough food sometimes or often and/or were unable to eat their preferred foods (Figure 1). Furthermore, 11 (2.8%) of the pregnant women were from moderate food unsecured households, where food quality was sacrificed more frequently by eating monotonous or undesirable foods and/or the food quantity was cut back on by reducing the size of meals or the number of meals, rarely or sometimes (Table 3).

**Table 3:** Household food security status of the pregnant women attending antenatal care at public hospitals in North Shewa, Oromia, Ethiopia, 2023 (*n* = 395).

Variables	Frequency	%
Worry about not having enough food		
Yes	198	50.1
No	197	49.9
How often does this happen (*n* = 198)		
Rarely	105	53.3
Sometimes	93	46.7
Unable to eat the kinds of foods you prefer		
Yes	140	35.4
No	255	64.6
How often does this happen (*n* = 140)		
Rarely	98	70
Sometimes	43	30
Eat a limited variety of foods or just a few kinds of foods		
Yes	56	14.2
No	339	85.8
How often does this happen (*n* = 56)		
Rarely	45	80
Sometimes	11	20
Eat foods you really do not want to eat		
No	395	100
Eat a smaller meal than needed		
No	395	100
Eat fewer meals in a day		
No	395	100
No food of any kind in the household		
No	395	100
Go to sleep hungry		
No	395	100
Go a whole day and night without eating		
No	395	100

### Pregnancy and eating habits-related characteristics

Approximately 200 (50.6%) of the pregnant women were in the second trimester of pregnancy during the assessment. More than one-third, 142 (35.9%), of the pregnant women reported that they had received counseling on dietary intake during their antenatal care visits from health professionals. Approximately 268 (67.8%) of the pregnant women consumed three or more meals in the last day before the assessment. Furthermore, 43 (10.9%) of the pregnant women fell sick 2 weeks before the date of the data collection (Table 4).

**Table 4:** Pregnancy and feeding pattern-related characteristics of the pregnant women attending antenatal care at public hospitals in North Shewa, Oromia, Ethiopia (*n* = 395).

Variables	Frequency	%
Gestational age		
First trimester	68	17.2
Second trimester	200	50.6
Third trimester	127	32.2
Illness in the last 2 weeks before the assessment		
Yes	43	10.9
No	352	89.1
The frequency of maternal food eating patterns		
Three or more meals per day	268	67.8
Two meals only or fewer per day	127	32.2
Dietary counseling during the ANC follow-up		
Yes	142	35.9
No	253	64.1

### Dietary diversity adequacy

**Figure 2:**
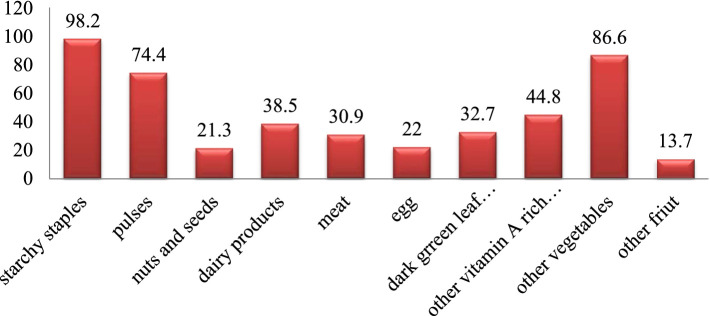
Dietary diversity adequacy of the pregnant women attending antenatal care at public hospitals in North Shewa, Oromia, Ethiopia, 2023 (*n* = 395).

Dietary diversity adequacy was determined by summing up the number of food groups consumed over a 24-h period by the pregnant women. According to the FAO individual and household dietary diversity guidelines, there are approximately 10 different food groups that determine dietary diversity. Each group was assigned a score of 1 if consumed and 0 if not consumed. Then, the scores were summed up for the food groups consumed and classified into inadequate dietary diversity, when the pregnant women reported consuming ≤4 food groups, and adequate dietary diversity, when the pregnant women reported consuming five or more food groups, out of the 10 food groups. The study found that the mean dietary diversity score among the pregnant mothers was 4.6 ± 1.12 standard deviations (SD). Among the total pregnant mothers, it was observed that 219 (55.4%) had adequate dietary diversity (dietary diversity score greater or equal to 5) and 176 (44.6%) had inadequate dietary diversity (dietary diversity score <4) in the previous 24-h recall periods. Figure 2.

Regarding the most consumed food groups by the pregnant women, almost all women, 388 (98.2%), ate starchy staple foods (grains, white roots and tubers, and plantains) and more than three-thirds of the pregnant women, 342 (86.6%), consumed other vegetables (onion, tomato, etc.) in the previous 24 h. On the other hand, eggs, 87 (22%), nuts and seeds, 84 (21.3%), and other fruits, 54 (13.7%), were the least consumed food groups by the pregnant women (Table 5).

**Table 5:** Dietary diversity adequacy of the pregnant women attending antenatal care at public hospitals in North Shewa, Oromia, Ethiopia, 2023 (*n* = 395).

Variables	Consumed/not consumed	Frequency	%
Grains, white roots and tubers, and plantains	Yes	388	98.2
	No	7	1.8
Pulses (beans, peas, and lentils)	Yes	294	74.4
	No	101	25.6
Nuts and seeds	Yes	84	21.3
	No	311	78.7
Dairy	Yes	152	38.5
	No	243	61.5
Meat, poultry, and fish	Yes	122	30.9
	No	273	69.1
Eggs	Yes	87	22
	No	308	88
Dark green leafy vegetables	Yes	129	32.7
	No	266	67.3
Other vitamin A-rich fruits and vegetables	Yes	177	44.8
	No	218	55.2
Other vegetables	Yes	342	86.6
	No	53	13.4
Other fruits	Yes	54	13.7
	No	341	86.3

### Factors affecting the dietary diversity adequacy of pregnant women

The association of the dependent and independent variables was explored by both bivariate and multivariate binary logistic regression analyses. The bivariate logistic regression analysis showed that place of residence, mother’s educational level, mother’s occupation, average household monthly income, having a latrine, practicing home gardening, frequency of meals per day, receiving dietary intake counseling during ANC, and no history of illness in the last 2 weeks before the assessment were the factors associated with the adequate dietary diversity of the pregnant women. Then, in the multivariable logistic regression analysis, some variables such as place of residence, mother’s occupation, average monthly income, practicing home gardening, and history of illness in the last 2 weeks before the assessment and receiving dietary intake counseling during ANC were factors associated with adequate dietary diversity.

The place of residence of the pregnant women was significantly associated with dietary diversity adequacy. The pregnant women who reported living in the urban area were 2.4 (AOR = 2.4, 95% 1.1, 5.18) times more likely to have adequate dietary diversity than those who reported living in the rural area.

Regarding women’s occupation, the pregnant women who were housewives (AOR = 2.5, 95% CI: 1.13, 5.58), were employed (AOR = 2.95, 95% CI: 1.17, 7.50), and were daily laborers (AOR = 3.57, 95% CI: 1.21, 10.57) were more likely to have adequate dietary diversity compared to those whose occupation was student, who were unemployed, etc.

The study also showed that the pregnant women whose average household monthly income was below 500 ETB (AOR = 0.25, 95% CI: 0.07, 0.83) were less likely to have adequate dietary diversity compared to those women whose average household monthly income was above 1,501 ETB. The pregnant women who reported practicing home gardening were 2.5 times more likely to have adequate dietary diversity when compared to those who reported not practicing home gardening (AOR = 2.5, 95% CI: 1.39, 4.5). Those who reported having three or more meals per day were three times more likely to have adequate dietary diversity in their diet compared to those who reported having two or fewer meals per day (AOR = 2.9, 95% CI: 1.64, 5.09). Similarly, the pregnant women who reported receiving dietary intake counseling during the ANC follow-up were 3.5 times (AOR = 3.56, 95% CI: 2.0, 6.35) more likely to have adequate dietary diversity (Table 6).

**Table 6:** Factors associated with dietary diversity adequacy of the pregnant women attending antenatal care at public hospitals in North Shewa, Oromia, Ethiopia, 2023 (*n* = 395).

Variables	Adequate dietary diversity		COR (95% CI)	AOR (95% CI)	*p*-value
	No	Yes			
Residence					
Urban	32	17	2.64 (1.412–4.94) *	2.4 (1.1, 5.18) **	*p* < 0.027
Rural	144	202	1	1	7
Mother’s educational status					
Unable to read and write	38	28	0.38 (0.19, 0.76) *	0.59(0.0.23, 1.54)	*p*-0.282
Able to read and write	40	57	0.74 (0.39, 1.38)	1.79 (0.73, 4.39)	*p-*0.203
Elementary school	43	38	0.46 (0.24, 0.88) *	0.99 (0.4, 2.50)	*p-*0.973
Secondary and preparatory school	29	46	0.83 (0.43, 0.16)	1.47 (0.63, 3.43)	*p-*0.374
College and above	26	50	1	1	
Mother’s occupation					
Housewife	102	122	2.51 (1.13, 5.58) *	3.44 (1.37, 8.68) **	
Employed	21	39	3.9 (1.55, 9.8) *	3.69(1.12, 12.16) **	*p*-0.032
Merchant	22	31	2.95(1.17, 7.50) *	3.43 (1.15, 10.24)**	*p*-0.027
Daily laborer	10	17	3.57(1.21, 10.57) *	3.66 (1.0, 13.45) **	*p*-0.050
Others	21	10	1	1	
Average monthly income					
Below 500 ETB	18	6	0.21 (0.08, 0.57) *	0.25 (0.07, 0.83) **	*p*-0.024
501–1,500 ETB	118	148	0.77(0.48, 1.23)	1.16 (0.63, 2.14)	*p*-0.64
Above 1,501 ETB	40	65	1	1	
Toilet					
Yes	148	206	2.99 (1.50, 5.98) *	0.77 (0.032, 1.84)	*p*-0.555
No	28	13	1	1	
Home gardening practice					
Yes	27	101	4.72 (2.89, 7.69) *	2.5 (1.39, 4.5) **	*p*-0.002
No	149	118	1	1	
History of illness in the last two weeks					
Yes	28	15	0.39 (0.20, 0.75) *	0.5 (0.23, 1.11)	*p*-0.089
No	148	204	1	1	
Mother’s meals in the last one week					
Three or more meals a day	88	180	4.62 (2.93, 7.28) *	2.9 (1.64, 5.09) **	*p* < 0.001
Two meals or fewer a day	88	39	1	1	
Dietary counseling during the ANC follow-up					*p* < 0.001
Yes	28	114	5.74(3.54, 9.30) *	3.56 (2.0, 6.35) **	
No	148	105	1	1	

*Significant at a *p* < 0.20. **Significant at a *p* < 0.05.

## Discussion

In this study, approximately 55.4%, with CI: 50.4–60.4%, of the pregnant women consumed a diet with adequate dietary diversity in the last 24 h. This proportion was lower than that of studies conducted in Laikipia, Kenya (60.6%) (30), and Ghana (85.5%) (31). The possible difference might be due to the difference in the study season and the sample size of the study participants. Furthermore, this study contained 10 food groups with two categories, whereas the study conducted in Ghana contained 11 food groups. In addition, geographical location, seasonal variability, and/or socio-cultural factors might have contributed to the difference in the results.

The result obtained in this study was also higher than those obtained in studies conducted in India (31.1%) (32), Burla (47.3%) (33), South Africa (25%) (8), Bale (44.8%) (27), Dire Dawa (43%) (18), and Alamata, Ethiopia (43.6%) (26). The difference might be due to the study period and the variations in the food group involved and its food category, as the study conducted in India contained eight food groups developed by the WHO and that conducted in Bale contained nine food groups. In addition, the variations in the geographical location and agricultural practice might be the reasons behind the difference in the results.

The results of this study indicated that nearly all (98.2%) of the pregnant women consumed grains, white roots and tubers, and plantains (starchy staple foods/cereals), while 86.6% of the pregnant women consumed other vegetables in the last 24 h, which was predominant. This study found that foods made from starchy ingredients and other vegetables, which may not provide a full range of micronutrients, were the study participants’ staple foods as they are locally grown and consumed by many respondents. This finding was almost consistent with the findings of other studies conducted in Vimsar town, Burla, which found that the most commonly eaten food groups were starchy staples (100%) and other vegetables (71.3%) (33), in Laikipia county, Kenya, (99.2% starchy staples and 92.9% other vegetables) (30), and in Dire Dawa city, Ethiopia, (100% starchy staples) (18). This indicates that the food consumption style in a resource-poor setting is almost similar as the people consume what is locally grown and easily available. This consistency could be explained by the fact that the study participants grow similar foods that are influenced by the same East African agricultural food production practices and geographical locations.

On the other hand, the nuts and seeds group (21.3%), egg group (22%), and other fruits group (13.7%) were the least consumed food groups by the pregnant women in the last 24 h. This finding indicated that the least consumed foods by the pregnant women are a good source of unsaturated fatty acid, vegetable and animal protein, vitamin B12, and other minerals. The finding was nearly consistent with the findings from previous studies conducted in Vimsar town, Burla, where other fruits (24%), eggs (14%), and nuts and seeds (8.7%) were the least consumed food groups (33). In addition, the study conducted in Dire Dawa city, Ethiopia, showed that eggs (16.3%) and other fruits (4.5%) were the least consumed food groups (18). A possible explanation for this finding might be that these food groups are often expensive and not accessible at an affordable price. In addition, a traditional way of farming results in less productivity and seasonal availability of nuts and fruits.

However, this finding was not in line with the study conducted in Kenya, where nuts and seeds were consumed by 54.3% of the study participants and other fruits by 51.3% of the study participants (30), and the study conducted in Shashemane, Ethiopia, where nuts and seeds were consumed by 58.7% and other fruits by 52.4% of the study participants (34). This difference might be due to the study period, geographical locations, and agricultural food production practice, which varied across different geographic locations.

The place of residence was significantly associated with adequate dietary diversity as the pregnant women who reported living in the urban area were 2.4 times more likely to have adequate dietary diversity than those who reported living in the rural area [AOR = 2.4, 95% CI: 2.4 (1.1, 5.18)]. This finding of the study was consistent with the study conducted in Bale zone, Ethiopia, which revealed that being a resident of the urban area made the participants 3.72 times (AOR = 3.72, 95% CI, 2.22, 6.20) more likely to have adequate dietary diversity than the participants residing in the rural area (27). This might be due to the lifestyle difference between the rural and urban areas, the availability of more packed and fresh foods in the urban area, and the geographic distance between rural households and the closest market where food can be bought or sold.

In this study, the occupation of the participants was found to be a predictor of dietary diversity. The women who were housewives, who were employed (both government and non-government), who were merchants, and who were daily laborers were more likely to have adequate dietary diversity compared to those whose occupation was student, who were unemployed, etc. This finding of this study was in line with the study conducted in Laikipia county, Kenya, which reported that being employed (salaried) had the highest odds (2.29 times) of attaining minimum dietary diversity as compared to the non-employed (AOR 2.29; 95% CI 1.18, 4.14) (30). This may be due to the fact that having a regular monthly income increases their chance of access to and choice of food. Another study conducted in Alamata General Hospital, Ethiopia, showed that being a government employee (AOR = 4.87, CI: 1.70–13.95) and a merchant (AOR = 4.67, CI: 1.81–12.05) were significantly associated with high dietary diversity (26). This could be explained by the fact that the pregnant women who were housewives reported to be responsible for buying and preparing meals while participating in market exchanges related to better living conditions.

This study also showed that dietary diversity was significantly associated with the monthly household income as with a decline in the pregnant women’s average monthly household income, the probability of consuming adequately diversified food was observed to decrease by 0.25 [AOR = 0.25, 95% CI: (0.07, 0.83)]. This result showed that a higher economic status was associated with higher dietary diversity adequacy. This finding was similar to the finding of an institutional-based study in Shashemane, Ethiopia, which showed that the pregnant women whose monthly household income was above 3,500 ETB had a high probability of attaining adequate dietary diversity compared to those whose monthly household income was <2000 ETB (34). This could be due to the fact that low-income earners are recognized to be negatively affected in their preference for the quality and quantity of diversified food groups consumed in their feeding arrangements, which is attributable to income.

The pregnant women who reported practicing home gardening were 2.5 times more likely to have adequate dietary diversity when compared with those who reported not practicing home gardening (AOR = 2.5, 95% CI: 1.39, 4.5). This finding was in line with the finding of the study conducted among pregnant women in Bale zone, Ethiopia, which revealed that pregnant women who practiced home gardening were 2.34 times more likely to have adequate dietary diversity when compared to those who did not practice home gardening (AOR = 2.34, 95% CI: 1.39, 3.94) (27). This could be explained by the fact that a home garden is a place from where household members can access a variety of foods and horticultural crops (tubers, vegetables, and fruits). In addition, a study conducted in Myanmar reported that access to a home garden was associated with a 7.7 percentage point lower probability of being in the lowest dietary diversity score category and a 13.1% point higher probability of being in the highest dietary diversity category compared to a household with no home gardens (35). This could be due to the reason that home gardens provide easy access to a variety of food that may not be available in the market through the cultivation of different vegetables, fruits, and other crops.

This study clarified that increasing meal frequency improves pregnant women’s dietary diversity, as those who reported having three or more meals per day were three times more likely to have adequate dietary diversity compared with those who reported having two or fewer meals per day (AOR = 2.9, 95% CI: 1.64, 5.09). This finding was consistent with the findings from a previous study conducted among pregnant women in East Gojjam zone, Northwest Ethiopia, which reported a lower likelihood of inadequate dietary diversity among women with increased meal frequency [AOR = 0.53, 95% CI (0.38–0.74)] (17). The finding of our study was also supported by a study conducted in Hosanna town, South Ethiopia. The study showed that pregnant women who consumed meals three times per day had 8.3 times (AOR = 8.3; 95% CI: 4.5, 15.6) greater odds of achieving adequate dietary diversity than those who had consumed meals two times per day (36). This could be explained by the fact that frequent, small, but balanced meals and three light snacks for pregnant women throughout the day increase the number of consumed food items and ensure that nutritional needs are met.

This study identified that the counseling on dietary intake during the ANC significantly increased the number of food groups consumed by the pregnant women (AOR = 3.56, 95% CI: 2.0, 6.35). This finding was supported by the study conducted on pregnant women in Hosanna town, South Ethiopia, which showed that pregnant women who received health education about the sources of foods containing iron, increasing meal frequency, and consuming diversified foods during pregnancy (AOR = 2.3; 95% CI: 1.2, 4.4) were 2.3 times more likely to have adequate dietary diversity as compared to those who did not receive health information (36). It was proven that integrating dietary counseling and nutrition intervention into an existing maternal, neonatal, and child health program significantly increased the number of food groups consumed by pregnant women in Bangladesh (37). This might be related to the fact that counseling during ANC facilitates effective communication with pregnant women about dietary diversity, which includes food sources of vitamins and minerals. In addition, it is the way of discussing locally held beliefs, attitudes, and misconceptions that prohibit pregnant women from consuming an adequate diversified diet during pregnancy.

## Limitations of the study

- The study failed to determine food intake in terms of the specific nutrients consumed.
- Being a cross-sectional study, the study might have limitations in reflecting the actual situation of seasonal differences in food availability in the study area.
- This study did not consider if there was any significant loss of nutrients during food preparation

## Conclusion and recommendations

This study showed that 55.4% of the pregnant women had adequate dietary diversity. Being an urban dweller, being a housewife, government employee, merchant, and daily laborer, having a lower household monthly income, practicing home gardening, having a frequency of three or more meals per day, and receiving dietary intake counseling during the ANC follow-up were positively associated with adequate dietary diversity.

- *Local microfinance and saving institutes*
- This study identified that the pregnant women from rural households and with low average monthly household incomes were more vulnerable to inadequate dietary diversity.
- Supporting and strengthening saving habits and establishing small-scale enterprises to create off-farm income opportunities for rural households can improve pregnant women’s purchasing power and lead to adequate dietary diversity.
- *Woreda agriculture offices and other non-governmental organizations*
- Promoting urban agriculture and home gardening practices among vulnerable households can improve dietary diversity among pregnant women.
- Increasing agro-biodiversity with production and productivity is crucial as more than one-third of the pregnant women were from a mildly food insecure household.
- *Health professionals and health extension workers*
- Appropriate dietary intake and meal frequency counseling during ANC, delivery, and postnatal care services are crucial to improve dietary diversity during pregnancy.
- *For researcher*
- Further research is recommended with a different study design (e.g., community-based) to address dietary knowledge, seasonal variability, and other variables that were not included in this study.
- More studies need to be conducted on the locally held beliefs, food taboos, and misconceptions toward diversified food intake during pregnancy in the study area.

## Data Availability

The raw data supporting the conclusions of this article will be made available by the authors, without undue reservation.
